# Melanin as a Photothermal Agent in Antimicrobial Systems

**DOI:** 10.3390/ijms25168975

**Published:** 2024-08-18

**Authors:** Arianna Menichetti, Dario Mordini, Marco Montalti

**Affiliations:** 1 Department of Chemistry “Giacomo Ciamician”, University of Bologna, Via Selmi 2, 40126 Bologna, Italy; arianna.menichetti2@unibo.it (A.M.); dario.mordini2@unibo.it (D.M.); 2 Department of Chemistry “Giacomo Ciamician”, University of Bologna, Tecnopolo di Rimini, Via Dario Campana 71, 47921 Rimini, Italy

**Keywords:** melanin, polydopamine, antibacterial properties, photothermal effect

## Abstract

Bacterial infection is one of the most problematic issues for human health and the resistance of bacteria to traditional antibiotics is a matter of huge concern. Therefore, research is focusing on the development of new strategies to efficiently kill these microorganisms. Recently, melanin is starting to be investigated for this purpose. Indeed, this very versatile material presents outstanding photothermal properties, already studied for photothermal therapy, which can be very useful for the light-induced eradication of bacteria. In this review, we present antibacterial melanin applications based on the photothermal effect, focusing both on the single action of melanin and on its combination with other antibacterial systems. Melanin, also thanks to its biocompatibility and ease of functionalization, has been demonstrated to be easily applicable as an antimicrobial agent, especially for the treatment of local infections.

## 1. Introduction

In the last few years, the massive use of antibiotics has led to the development of bacterial resistance, which today represents one of the major threats to human health [1]. Indeed, bacterial and fungal proliferations can crucially reduce the quality of life of human beings; for example, septicemia and other microbial-related diseases are still among the major causes of deaths worldwide [2,3]. Bacteria have developed many methods to resist to antibiotics, such as the expression of proteins that promote drug alteration [4] or of genes that modify the binding surface targeted by the drug [5], and bacteria biofilms, which, by means of their organization, provide more effective defense mechanisms [6,7]. This bacterial evolution has made urgent the need for new antibacterial agents, which could overcome the resistance to antibiotics. Among these new agents, nanomaterials are widely used. Indeed, nano-sized systems are largely employed in biomedicine since their size allows for efficient internalization through cell membranes and their surface enables functionalization with targeting moieties and encapsulation of drugs [8,9,10,11,12]. These features make nanomaterials optimal candidates for antibacterial therapy. Recently, melanin has been explored as a new polymeric material with many applications in nanomedicine [13,14]. Indeed, melanin, which can be extracted from natural sources (such as cuttlefish ink) or synthesized (polydopamine—PDA) has been reported to have an excellent biocompatibility [15], which is one of the reasons for its use in biomedicine. Additionally, melanin-like materials are mostly extracted or synthesized as nanoparticles, with many functionalities on the surface (catechol, amine and imine) that allow for the combination with other agents, such as drugs or targeting molecules [16]. Apart from these features, melanin-like nanomaterials also have antibacterial properties, which are well described by Fu et al. [17]. They have individuated four main antibacterial effects of polydopamine. First, it can interact with the bacterial cell membrane, with the chelation of ions or proteins and with electrostatic forces, provoking bacteria destruction [18]. Secondly, the amine groups of PDA can be functionalized with halogen atoms, leading to N-halamine groups, which have high antimicrobial properties [19]. Also, PDA can generate reactive oxygen species (ROS) by means of the catechol groups that transfer electrons with the phenolic quinone isomerism [20]; ROS formation obviously contributes to bacterial death. Finally, PDA antibacterial activity is due to its photothermal properties.

The photothermal behavior of melanin has been widely explored for photothermal therapy: the fast heat increase disrupts the cytoplasmatic components of cancer cells, leading to cell apoptosis [21]. The destruction of bacteria with a photothermal effect occurs with similar mechanisms and opens up the possibility of the large-scale use of this material in antimicrobial applications. For this reason, the photothermal effect represents the most investigated antimicrobial mechanism induced by melanin.

In this review, the recent advances in the antibacterial use of the photothermal properties of melanin will be described. Systems in which the antimicrobial activity is due to the photothermal effect of melanin-like materials are reported, particularly for local infections, such as wound healing. Indeed, here melanin is activated by light, which can act with very high precision, just within a specific area. This makes the photothermal technique especially promising for local treatments. However, often, melanin action is not sufficient to completely eradicate bacteria and needs to be coupled with the effects of other agents [22]. Thus, here, we will distinguish systems in which the antibacterial activity is only provided by the presence of melanin and systems in which the synergy between melanin and other molecules or material is essential to enhance the antibacterial performance.

## 2. Melanin Photothermal Properties for Antibacterial Activity

**Figure 1:**
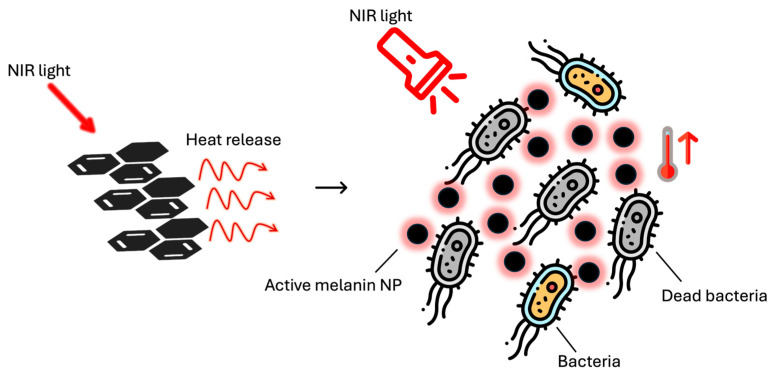
Mechanism of the photothermal effect in melanin NPs and its use in the eradication of bacteria through light-induced fast heat release.

Melanin (from the ancient Greek word μέλας (mèlas = black)) is the name of a class of black, brown and reddish polymeric bio-pigments that are mainly responsible for (i) photoprotection of biological tissues, (ii) pigmentation and structural coloration of skin, hair and feathers, (iii) antioxidant activity against ROS and (iv) antibacterial properties [23]. Melanin can be divided in sub-classes depending on the organism of origin or the chemical precursor [24]. Among animals, melanin is generally biosynthesized through tyrosine metabolism by melanocytes, ending up being one of the main components of the melanosomes, and it happens in two different forms: eumelanin and pheomelanin [25]. The former is characterized by a brown–dark color, while the latter displays more reddish nuances. In plants and fungi, melanin is often a nitrogen-free biopolymer rich in polyphenols, which is named allomelanin [26]. However, an overall characterization of the biochemical synthetic paths of melanin is still missing and their actual chemical structure is still unclear [27]. Over the years, several protocols have been optimized to synthesize biomimetic melanin materials in the laboratory. Indeed, eumelanin-like materials can be artificially obtained by stirring an alkaline aqueous solution of dopamine; for example, Ju et al. reported the formation of a colloidal solution of PDA NPs by mixing dopamine in an NaOH/water solution [28], while Xiao et al. reported the self-assembly of PDA NPs combining dopamine with an NH_3_/ethanol/water mixture [29]. As already mentioned, the extensive use of pesticides and drugs in the last few decades has driven the development of antibiotic resistance in several bacterial strains, reducing the effectiveness of several pharmaceutical therapies [30,31]. To overcome this issue, thermal therapy can be a winning solution; indeed, the overheating treatment is efficient over a wide range of microorganisms, without inducing metabolic immunity toward it [32,33,34]. For example, most *Escherichia coli* (*E. coli*) strains display gene disruption or overexpression when exposed to temperature higher than 60 °C [35]. Because of that, it should not be surprising that in the last few years, photothermal therapy has emerged as a very promising solution [36,37] (Figure 1).

**Figure 2:**
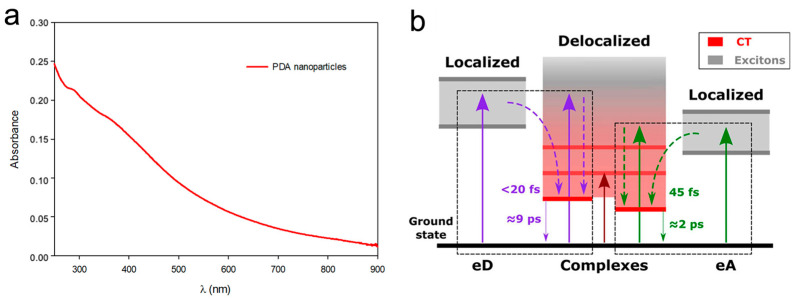
(**a**) Absorption spectrum of an aqueous solution of PDA NPs in the UV-vis-NIR region (from 250 nm to 900 nm). (**b**) Simplified scheme of the electronic states and transitions involved in PDA. The diagram describes the localized electronic states of electron-donor chromophores (eD) and electron-acceptor chromophores (eA), which compose the PDA structure. The coupled complexes (PDA) display substantial charge transfer character (CT) (red region), while the excitonically coupled or uncoupled chromophores result in neutral delocalized excitonic contributions (gray region). The complexes’ electronic states deactivate in less than 10 ps. Adapted with permission from ref. [46] Copyright 2024 American Chemical Society.

This technique exploits the release of heat when the molecular excited state undergoes non-radiative decay [38]. Indeed, with this method it is possible to increase the temperature in a confined space simply by light irradiation and its performance depends on the conversion efficiency of light to sufficient heat with photothermal nanomaterials [39]. In general, an adequate photo-absorber is injected, or it is directly applied onto the injured spot. Then, the photothermal material is activated by an external light source, with the aim being to generate hyperthermia in a diseased tissue. The confinement of the heating to a single location is crucial because it allows targeting of just the site of interest, reducing the damage to the healthy flesh [40]. For this purpose, the activation of a material through luminous stimulus is of great interest because it allows great control in space and time, which is crucial in the treatment of diseases like wounds [41]. For this aim, the best candidates are materials with a very low luminescence quantum yield and with a high absorption coefficient all over the UV-vis-NIR spectrum, like melanin [42]. Indeed, its structure, composed of oligomers conjugated and aggregated by π–π stacking interactions [43], promotes very efficient heat release after light excitation [42]. Indeed, it is well known that eumelanin is an engineered material that acts as a shield against the UV light and its adverse effects in many organisms, including human beings [44]. This is because natural- and biomimetic-melanin nanoparticles, like PDA, are characterized by broad-band monotonic absorbance, which guarantees a screening effect over a wide range of wavelengths (Figure 2a) [28,45]. Moreover, once excited, the different chromophores that compose the complex structure of such melanin de-activate in few pico-seconds in a non-radiative way, releasing heat (Figure 2b) [46]. For application in nanomedicine and for antibacterial systems, melanin activation is usually triggered by irradiating with NIR light, which is less energetic and the most transparent to biological tissues. The conversion from light energy into thermal energy is very efficient (40%); for example, after irradiation for 500 s, the temperature of a PDA NPs aqueous solution has reported to be increased by 33.6 °C at a concentration of 200 μg mL^−1^ [42].

## 3. Antibacterial Systems Based on Melanin Action Only

As already mentioned, the photothermal properties of melanin, coupled with its excellent biocompatibility and ease of functionalization, make it a proper candidate for antibacterial applications. However, based on the specific application, the use of melanin, which is usually in the form of NPs, needs support for its antibacterial function. For example, a common application of antimicrobial agents is wound dressing. Here, the antibacterial agent should be embedded in a soft material, which can easily fit wounds with irregular shapes, with tunable physical and chemical properties [47]. For this reason, hydrogels are often employed for application in wound healing [48]. Indeed, melanin used for antimicrobial purposes is often embedded in these gel matrixes. In this context, a very interesting recent review by Xu et al. reports the several couplings between melanin and hydrogels [49]. Indeed, melanin NPs not only confer photothermal and antioxidant properties to the system but can also provide network crosslinking points in the gel structure and increase its adhesive properties, thanks to their surface functional moieties. Other common platforms for antibacterial agents are films. They can be employed for multifunctional devices that require antibacterial protection [50], such as food packaging [51] and medical and personal electronic devices [52]. In Section Recent Advances in the Use of Melanin as an Antibacterial Agent, we describe some recent papers about the use of melanin NPs as antibacterial agents, which are incorporated in structures as hydrogels or films, mainly employed in local infection treatments.

### Recent Advances in the Use of Melanin as an Antibacterial Agent

**Figure 3:**
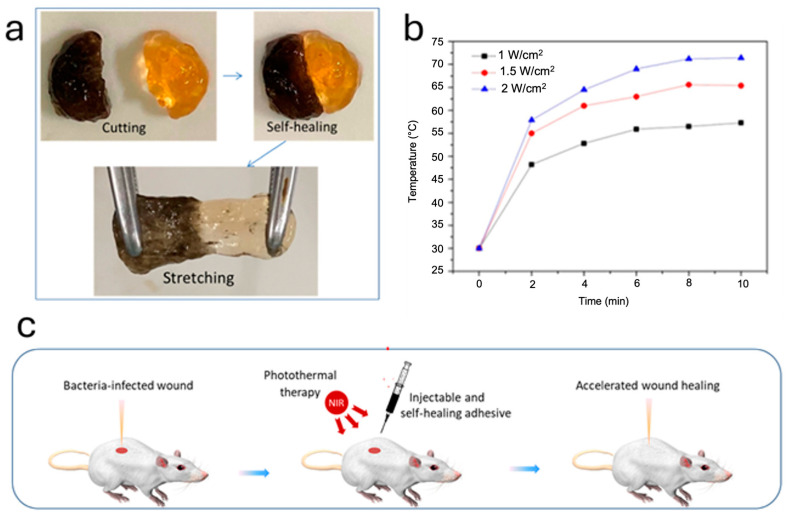
(**a**) Demonstration of the self-healing properties of the PDA@GC hydrogel. (**b**) Temperature changes in the hydrogel with varying power densities of an 808 nm laser. (**c**) Schematic representation of the infected wound-healing treatment. Adapted with permission from ref. [53] Copyright 2022 American Chemical Society.

Herein, we are going to present recent studies about biomimetic melanin materials and how their photothermal action can efficiently eradicate bacterial contaminations. Indeed, PDA has been integrated in several biomaterials with the aim of performing photothermal disinfection against microbes, bacteria and fungi. To begin with, Guo et al. developed a multifunctional injectable adhesive hydrogel with self-healing capacity and excellent photothermal antibacterial activity to promote bacteria-infected wound healing (Figure 3a) [53]. PDA NPs were incorporated into the hydrogel through Schiff base reactions between the quinone group on PDA NPs and the primary amine in glycol chitosan (GC). Upon NIR laser irradiation (808 nm, 1.0 W cm^−2^), the hydrogel temperature increased up to 57.9 °C (Figure 3b); indeed, as we are going to see in the next example, irradiation at 808 nm is often performed. The hydrogel showed excellent antibacterial activity under NIR irradiation for 10 min, with a killing rate of 98.2% for *E. coli* and 73.1% for *Staphylococcus aureus* (*S. aureus*). Hence, the wounds treated with the hydrogel and NIR irradiation showed a smaller dermal gap, more blood vessels, more sebaceous glands, and more hair follicles compared to the untreated ones (Figure 3c).

The processability of PDA in different morphologies has inspired researchers to develop technologies going beyond the NP form. For example, Huang et al. designed a self-healing hydrogel on the basis of quaternized chitosan (QCS), oxidized dextran (OD), tobramycin (TOB), and PDA-coated poly-pyrrole nanowires (PPY@PDA NWs) to improve burn wounds’ healing [54]. Indeed, the incorporation of PPY@PDA NWs endowed the hydrogel with near-infrared (NIR) irradiation-assisted bactericidal activity of drug-resistant bacteria, conductivity, and antioxidant activity. Accordingly, it was demonstrated that after adding PPY@PDA NWs, the photothermal effect of the hydrogel was rapidly improved and its temperature was increased up to 21 °C after 10 min of NIR irradiation. The hydrogel with a good photothermal effect achieved a sterilization ratio against *Pseudomonas aeruginosa* of 98% after only 1 min of NIR irradiation. Indeed, burn wounds treated with the hydrogel and NIR light demonstrated a faster wound contraction and better healing compared to untreated lesions. Following a different path, Li et al. polymerized dopamine (DA) directly into a cryogel matrix using NaIO_4_ [55]. As in the previous cases, the material demonstrated excellent photothermal properties by increasing the local temperature until 58.6 °C under NIR irradiation (880 nm) and good disinfection capability against *E. coli* and *S. aureus*.

Apart from PDA, other kinds of melanin have been applied as promising photothermal nanomaterials against pathogenic microorganisms. For example, Li et al. designed an injectable adhesive based on adipic dihydrazide-modified hyaluronic acid and benzaldehyde group-functionalized poly(ethylene glycol)-co-poly(glycerol sebacate), which has been doped with cuttlefish-derived melanin nanoparticles, also known as *Sepia* melanin [56]. Xiang et al. incorporated poly-1,8 dihydroxynaphtalene NPs (p-1,8DHN) in a polysaccharide-based hydrogel [57]. p-1,8DHN is a biomimetic analogous of allomelanin, a bio-pigment that can be found in fungi and plants [58]. Interestingly, in this case, upon NIR irradiation (880 nm), the hydrogel temperature increased up to 80.8 °C, eradicating almost 100% of the microbes. These examples highlight the potential use of different kinds of melanin in the treatment of local infections, thanks to its excellent photothermal performance, which allows it to act with high precision in the area of interest.

## 4. Antibacterial Systems Based on the Synergy of Melanin and Other Agents

As previously described, melanin has intrinsic antibacterial properties, which can be employed in several applications. However, its antibacterial action, which in this review is focused on its photothermal effect, is often not sufficient to efficiently eradicate bacterial colonies. Indeed, the application of a single compound might not be enough to fight highly resistant pathogens; thus, the application of a multi-component material is required [59,60]. Consequently, research has been focused on the coupling of melanin with other antibacterial agents to potentiate the antimicrobial action. There can be diverse strategies to increase the antibacterial performance. In particular, melanin can be coupled with agents that can push its antibacterial activity. This happens, for example, in melanin’s interaction with a system that could enhance the photothermal properties of melanin and its photostability. Examples include metals such as silver, gold or copper, whose binding to melanin NPs is favored by its multiple functionalities [17,61]. Often, these ions (silver ions, for example) also present their own antibacterial activity, contributing to the higher performance of the whole system. Another strategy is to couple melanin’s photothermal effect with the release of antibiotic drugs to obtain multiple antimicrobial therapy. This is also possible due to the various binding possibilities that melanin offers. In Section Composite Nanomaterials for Improving Antimicrobial Activity of PDA, we will summarize some of the recent advances in the synergistic effect of melanin and other agents to enhance the antimicrobial effects.

### Composite Nanomaterials for Improving Antimicrobial Activity of PDA

**Figure 4:**
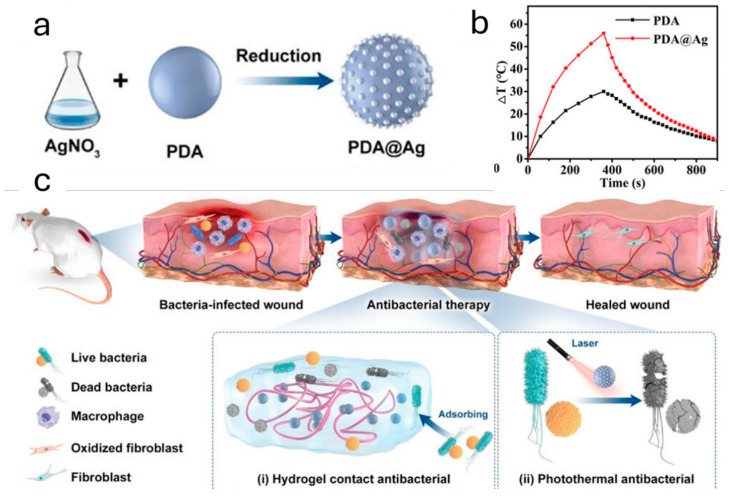
(**a**) Schematic synthesis of PDA@Ag NPs and (**b**) their photothermal performances compared to PDA NPs upon light irradiation at 808 nm. (**c**) Schematic representation of the infected wound-healing treatment: (**i**) adsorption of bacteria onto the hydrogel (**ii**) and its photothermal effect against microbial infection under laser irradiation. Adapted with permission from ref. [62] Copyright 2022 Wiley-VCH GmbH.

Here, we are going to present how PDA can work synergistically with other materials to increase the chance of success regarding disinfection treatments against harmful microbes. Researchers have developed several strategies to improve the photothermal properties and photostability of PDA by coupling it with other materials. For example, Qi et al. demonstrated that under NIR irradiation at 808 nm, the photothermal conversion efficiency of their PDA NPs and Ag-doped PDA NPs (PDA@Ag NPs) was 16.6% and 36.1%, respectively (Figure 4a,b) [62]. As a consequence, upon irradiation (808 nm, 1.0 W cm^−2^), a PDA NPs-doped cationic guar gum (CG) hydrogel reached a temperature of 46.4 °C in 3 min, while under the same conditions, a CG hydrogel doped with PDA@Ag NPs reached a temperature of 56.2 °C. Furthermore, Ag NPs are well known to be excellent antibacterial nanomaterials because of their Ag^+^-releasing properties [63]. Indeed, in 12 days, an infected wound treated with PDA NPs-doped hydrogel and NIR irradiation (808 nm, 1.0 W cm^−2^) was still 23.9% open with respect to the original lesion, while the one treated with PDA@Ag NPs-doped hydrogel and NIR irradiation was just 3.9% open (Figure 4c).

**Figure 5:**
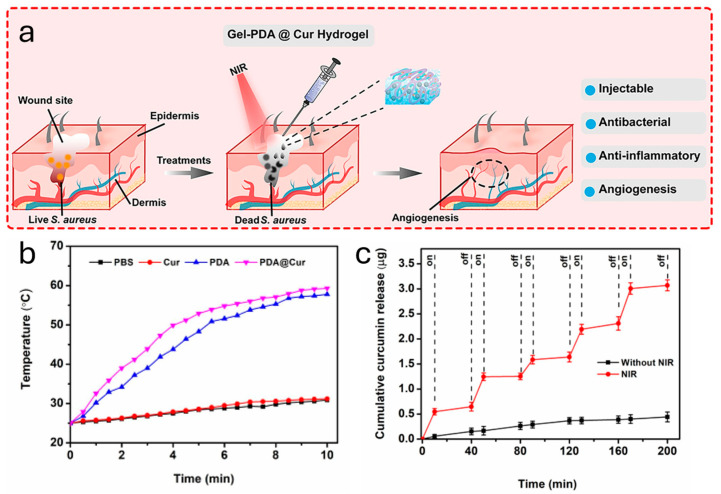
(**a**) Schematic representation of the application of PDA NPs@Cur hydrogel upon NIR irradiation on a microbial infected wound and its successful use. (**b**) Temperature increment upon NIR laser irradiation (808 nm laser irradiation (1.0 W/cm^2^)) of several solutions containing phosphate-buffered saline (PBS), Cur, PDA and PDA@Cur. (**c**) Cur release profile from the hydrogel with/without NIR laser irradiation (808 nm laser irradiation (1.0 W/cm^2^)). Adapted with permission from ref. [69] Copyright 2020 Elsevier B.V.

It is interesting to note that the same effect can be obtained by employing other metals, like Cu. In fact, Xu et al. designed a multifunctional composite hydrogel mainly composed of PDA and copper-doped calcium silicate ceramic (Cu-CS) to treat infected wounds [64]. Upon NIR irradiation (808 nm, 0.8 W·cm^−2^ for 5 min), the Cu^2+^ ion-doped hydrogel reached a temperature of 60 °C, while the control sample, which contained only PDA, reached a temperature of 42 °C. Indeed, the former material showed an *E. coli* bacterial inhibition percentage of almost 96%, while for latter one, it was almost 49%, once irradiated for 15 min. The superior properties of PDA@Cu composites were also exploited by other researchers. Indeed, Xia et al. presented a photothermal polyvinyl alcohol (PVA)-based hydrogel membranes with copper ion-loaded polydopamine to simultaneously achieve antibacterial functions in a wound dressing [65]. Indeed, PDA chelates Cu strongly because of their catechol units. In this case, the temperatures of the PVA/PDA and PVA/PDA@Cu rapidly increased to more than 50 °C within 5min after NIR irradiation (808 nm 2.0 W/cm^2^). In addition, the introduction of Cu further increased the photothermal efficiency of the hydrogels and the temperature of PVA/PDA@Cu reached 55–60 °C. The PVA/PDA@Cu hydrogel showed a great antibacterial effect because of the synergetic effect of photothermal therapy and the Cu-induced antibacterial effect. As a result, up to 99% of *E. coli* and *S. aureus* were killed. However, the authors stressed that the Cu concentration in the hydrogel should not overcome a threshold value (in this case >10 mM) because it could induce cytotoxicity in healthy tissues. Another winning solution to enhance the antimicrobial activity of PDA composite materials is to extend their photostability after several ON/OFF irradiation cycles. For example, Guo et al. proposed a hydrogel composed by polyacrylamide (PAM) and PDA, with the addition of Mg^2+^ ions [66]. The PDA/PAM/Mg^2+^ hydrogel showed a higher photothermal stability than the PAM/PDA hydrogel; in fact, after four on/off cycles, PAM/PDA could only induce an elevated temperature of 14.3 °C, lower than that of PDA/PAM/Mg^2+^, which was 21.3 °C. It is interesting to note that the survival rate for *S. aureus* was almost 77% and 75% for *E. coli* in a bacterial culture treated with PDA/PAM/Mg^2+^ hydrogel in the absence of NIR irradiation. In contrast, almost 93% of *S. aureus* and 94% of *E. coli* were alive after treatment with PAM/PDA hydrogel under the same conditions; this suggests that Mg^2+^ ions themselves display antimicrobial effectiveness. Moving on to a different aspect, it is interesting to note that different materials can have different antimicrobial properties following different antimicrobial mechanisms; indeed, photothermal treatment is just one of the possible strategies that can be used to eradicate microbial contaminations. This means that by employing more than one active substance at once, it is possible to tackle microorganisms from different routes, improving the performance of the treatment. For example, Xiao et al. encapsulated vancomycin (Van), a potent antibiotic, inside the porous structure of a zeolitic imidazolate framework-8 (ZIF-8), which has been coated with PDA [67]. The material exhibited a photothermal conversion of ~38% under NIR irradiation (808 nm). Also, the temperature increment enhanced the drug viability; indeed, at pH 4.7, the levels of released Van were ~37% in the dark, while upon NIR irradiation, they increased to almost 65%. PDA coating and the photothermal properties used to design a light-controlled method for the drug release were also exploited by He et al. by covering Au nanorods (GNRs) with a PDA shell [68]. The PDA coating acquired a high loading efficiency for daptomycin (DAP) because the hyperthermia could trigger more DAP release, resulting in further improvement to the bactericidal efficiency. Once the DAP-GCS-PDA@GNRs suspensions were irradiated with the 808 nm laser (0.5 W/cm^2^), the temperature increased promptly within 4 min, and it showed the highest temperature of about 60.7 °C with the concentration of 47 g/mL. Indeed, the photothermal conversion efficiency of the composite nanomaterial was calculated as ~27%. This biomaterial was applied on mice for the disinfection of subcutaneous abscesses. After injection, the implantation of DAP-GCSPDA@GNRs and NIR irradiation (0.5 W/cm^2^, 7 min), the temperatures of both the abscesses rapidly increased to ca. 50 °C. After NIR irradiation, DAP-GCSPDA@GNRs showed a strong antibacterial efficacy, with a 98.5% reduction in the bacterial colony at the infected site. Previously, we talked about PDA NPs with a full inner core, without any cavities in their structure. However, it is possible to synthesize PDA NPs with an intrinsic porosity, the so-called mesoporous poly-dopamine nanoparticles (MPDA NPs). Because of their spongy textures, MPDA NPs showed a higher drug load capability with respect to PDA NPs [14]. Indeed, a synergistic therapy based on antibiotics and the photothermal effect can be designed, like in the case of Tao et al. [69]. They presented a hydrogel that was composed of dibenzaldehyde-grafted poly(ethylene glycol) (PEGDA), lauric acid-terminated chitosan (Chi-LA), and curcumin (Cur)-loaded mesoporous polydopamine nanoparticles (PDA NPs@Cur) to promote infectious wound healing (Figure 5a). Indeed, Cur has been widely studied because of its therapeutic properties. In this case, upon NIR irradiation, the photothermal effect of the hydrogel not only generated local hyperthermia for killing bacteria but also promoted higher Cur release (Figure 5b,c). Indeed, the NIR-irradiated biomaterial demonstrated a killing rate higher than 90% against *E. coli* and *S. aureus*. Thus, the application of the hydrogel on a dermal lesion brought about an improvement in the antibacterial ability, anti-inflammatory property, collagen deposition, and granulation tissues under NIR irradiation.

PDA has also been combined with other photothermal organic materials, like graphene oxide, to further improve the light-induced heating process. An interesting example was provided by Zhang et al. [70]. Herein, the authors designed a polymeric film based on PDA, GO and polyethyleneimine (PEI), which had outstanding antimicrobial effectiveness against Gram-positive and Gram-negative bacteria upon irradiation by a NIR laser. The GO-PDA-PEI membrane demonstrated heating to about 67 °C after 3 min of NIR laser (795 nm) irradiation. Notably, the photothermal effect exhibited outstanding antibacterial activity (over 99%) for both *S. aureus* and *E. coli*. Finally, it is interesting to discuss the method of implementing the adhesion between PDA-based materials and bacteria to improve the efficiency of the treatments. Indeed, PDA NPs have a negatively charged surface that is electrostatically repulsed by the negatively charged lipidic membrane of numerous bacteria [71]. However, the grafting of charged functional groups on the surface of PDA-based materials can improve the adherence with biological matter [72]. This strategy was followed by Wu et al.: they decorated Rose Bengal (RB)-PDA NPs in a layer-by-layer fashion with polymyxin B (PMB) and gluconic acid (GA) to generate photoactive NPs (RB@PMB@GA NPs) for the treatment of infected wounds [73]. The amine groups of PMB can be easily pronated; in fact, for pH < 5, the RB@PMB@GA NPs showed positive Z-potential values. As they expected, the RB@PMB@GA NPs showed outstanding photodynamic antibacterial properties against *E. coli* at pH 5.0, while the RB@PMB@GA NPs did not exhibit any bacterial killing effect on *E. coli*, with or without irradiation, at pH 7.4.

## 5. Future Challenges

In the previous sections, we reported the use of melanin-based materials for the implementation of new antibacterial methods based on the treatment of local infections, exploiting melanin’s photothermal properties. This is an application in which research on melanin is continuously evolving and still requires some improvements.

### 5.1. Further Exploration of Melanin’s Properties

One of the main aspects that could be investigated in the future is the exploitation of all melanin’s multiple properties that could be useful for antibacterial purposes. At the beginning of this review, we described the basis of melanin’s antimicrobial activity: surface chelation of ions and binding to the bacterial surface, functionalization of amine groups with halogens, ROS production, and photothermal properties. Among these possibilities, the most investigated one concerns the photothermal effect, which, however, is sometimes not sufficient to achieve effective bacteria destruction and melanin often needs to be coupled with other agents. According to this point, it would be interesting to exploit the different antibacterial actions of melanin in the same system and at the same time. Indeed, melanin could intrinsically represent a multiple antibacterial agent, without the need for external agents.

### 5.2. Surface Modification of Melanin for Better Interaction with Microorganisms

Melanin’s surface functionalization is one of the main advantages of this material. Melanin’s surface charge, at a neutral pH, is negative, because of the deprotonation of the catechol moieties [74], but the surface charge can be modulated by the attachment of other functional groups. The possibility of tuning the surface charge of melanin can increase the interaction with the external membranes of both Gram-positive and Gram-negative bacteria, thus improving the antibacterial properties with a selective strategy. Thus, a deeper study on the tuning of melanin NPs’ zeta potential could be beneficial to engineer a system with maximal interaction with the targeted microorganisms.

### 5.3. Biocompatibility of Composite Systems

In many cases, the antibacterial activity is based on the synergic action of different agents, and this is mainly true for melanin, whose single activity seems not to be sufficient. Thus, there are a lot of composite systems for antimicrobial purposes. However, when the applications are medical devices or wound healing, it is necessary to preserve the biocompatibility of such complex materials. For example, when there is delivery of drugs or metal ions, further investigation on the more controlled and selective release of these agents should be essential to provide a safer application.

### 5.4. Scale-Up: A Problem Related to Costs, Drug Administration Agencies’ Approval and Applications on Real-World Problems

Academic research is fundamental to better understand the surrounding world and to develop new technologies that can have a huge impact on human life. However, winning solutions in the lab can fail in relation to real-world problems. Indeed, the transposition of promising research results into an industrially scalable process is full of obstacles, like costs, legislative rules and marketing requirements. Functional materials and sophisticated instrumentation can work, but their cost is often prohibitive for companies, especially middle–small ones. In addition to that, therapeutic and non-therapeutic drugs must be approved by competent bodies like the Food and Drug Administration (FDA). To date, the agency has not officially defined the universal criterion for the term antibacterial; however, based on the last guidelines issued to industrial companies, the agency considers > 3 log (>99.9%) proliferation reduction in microbial colony-forming units against the worst-case (i.e., resistant forms) strains of bacteria of at least three Gram-positive and three Gram-negative bacteria that are related to the intended use (e.g., dermal wound infections) as effective antibacterial activity [75]. As we presented in Section 3 and Section 4, most of the proposed technologies cannot satisfy the antimicrobial standards that are usually needed to be certificated by FDA. For this reason, a further implementation of the treatment performances is required. Finally, it is necessary to highlight that academic studies rely on standard models. In medicine, every single patient and every single disease has its own peculiarities. For example, wounds can occur in multiple shapes, dimensions, depths, infection severities and locations. Because of that, the effectiveness of therapy can depend on the wound’s properties. This means that a therapy can be winning for a specific wound, but it cannot be considered so for a wound of the same kind but with different characteristics. Indeed, further investigation on infected substrates with different characteristics should be pursued to validate the cure.

### 5.5. Adverse Reaction to the Application of Photothermally Active Material for Human Health

Despite the extensive investigation of photothermal agents in the biomedical field for the treatment of cancer and infected wounds, this kind of therapy can suffer from several adverse reactions. For this reason, the application of photothermally active compounds is still limited. One of the major problems is the unintentional damage to the tissues of the treated area because of hyperthermia. Indeed, a proper photothermal treatment should kill the bacteria without any damage to the tissue. This issue could be solved with a higher control of the temperature, also considering the variety of the tissues that could be damaged and their thermal conductivity. In particular, the melanin-based material would need to be engineered to precisely correlate the light power to the temperature obtained, using lower temperatures when needed. Another strategy could be to induce the temperature increase in short time intervals, since short heating times allow for higher tissue preservation than long heating times [76]. Moreover, damage to healthy tissues nearby to the diseased spot could also occur because the photothermal agents may lack a selective accumulation in the infected regions, and they can diffuse in their proximity. In this case, hyperthermia could be induced even in normal tissues, causing involuntary damage. To avoid this side effect, it is necessary to improve the targeting ability of the photothermal agent [77]. In addition to that, the local heat release can induce localized pain in the treated area, hampering the healing process. Indeed, the constant conversion of NIR light can overheat the normal tissues close to the infected target, causing inflammation and pain perceived by the patient. Moreover, to achieve complete eradication of bacteria, a high-power light source may be needed to increase the temperature over 50 °C, which might be painful for the patient. In the case of skin treatment, some strategies can include topical pre-medication with lidocaine, prilocaine gel, injection of topical anesthetics, cooling with cold air, applying nerve blocks, and pulsed laser irradiation [78]. Indeed, the application of these side treatments should be considered when clinical tests are performed to evaluate the performances of antimicrobial materials.

## 6. Conclusions

Melanin is one of the most investigated materials, with applications in many fields, from nanomedicine to cosmetics and electronics, because of its outstanding properties, which confer great versatility to this material. Lately, the antibacterial properties of melanin have been widely investigated, based on melanin’s surface properties and ROS generation, but mainly on its photothermal behavior, which can destroy microorganisms with light irradiation. Many systems based on this effect have been developed, using only the action of melanin or combining melanin to potentiate the antibacterial effect. These systems already show very good performances against microbes, and melanin, if properly engineered to exploit all its properties to a maximum, could be even more commonly employed for the generation of new antimicrobial systems, contributing to solving the issue of antibiotic resistance.
